# Adaptation of HIV-1 Depends on the Host-Cell Environment

**DOI:** 10.1371/journal.pone.0000271

**Published:** 2007-03-07

**Authors:** Tim van Opijnen, Anthony de Ronde, Maarten C. Boerlijst, Ben Berkhout

**Affiliations:** 1 Department of Human Retrovirology, Academic Medical Center, University of Amsterdam, Amsterdam, The Netherlands; 2 Section Population Biology, Institute for Biodiversity and Ecosystem Dynamics, University of Amsterdam, Amsterdam, The Netherlands; National Cancer Institute at Frederick, United States of America

## Abstract

Many viruses have the ability to rapidly develop resistance against antiviral drugs and escape from the host immune system. To which extent the host environment affects this adaptive potential of viruses is largely unknown. Here we show that for HIV-1, the host-cell environment is key to the adaptive potential of the virus. We performed a large-scale selection experiment with two HIV-1 strains in two different T-cell lines (MT4 and C8166). Over 110 days of culture, both virus strains adapted rapidly to the MT4 T-cell line. In contrast, when cultured on the C8166 T-cell line, the same strains did not show any increase in fitness. By sequence analyses and infections with viruses expressing either yellow or cyan fluorescent protein, we were able to show that the absence of adaptation was linked to a lower recombination rate in the C8166 T-cell line. Our findings suggest that if we can manipulate the host-cellular factors that mediate viral evolution, we may be able to significantly retard viral adaptability.

## Introduction

Disease progression in HIV-1 infected patients shows extensive variation; some patients efficiently suppress HIV-1 for more than 15 years while others develop AIDS within several years [1], [2]. Disease progression is related to the viral set-point and viral load, and patient to patient variability can be explained, to some extent, by the efficacy of the host-immune response [3], which is influenced by *e.g.* the CD4+repertoire, CD8+repertoire and antibodies [4]. Without intervention, the virus almost always brakes through the host defenses and the patient will eventually develop AIDS. One of the most important and hard to battle characteristics of HIV-1 is its large adaptive potential. It is this potential that makes the virus escape from the host-immune system and resistant to anti-retroviral drugs. Several viral-characteristics that contribute to HIV-1′s adaptive potential are its short generation time [5], [6], high copy numbers [7], [8], and high mutation rate [9]. In addition, recombination may be important, for example, in combining resistance mutations into a single genome to achieve drug-resistance [10], [11]. The host may also affect the adaptive potential of the virus. For instance different target cell environments support differential viral replication rates [12], generation time [13] and recombination rates [14]. Thus, the adaptive potential of HIV-1 is both shaped by the virus and the interaction between the virus and the host-environment. To which extent the host affects the adaptability of HIV-1, however, is still largely unexplored.

One viral trait that contributes to an unknown degree to the adaptive potential of HIV-1 is viral recombination. For effective recombination to occur in HIV-1, a single host-cell needs to be infected with two distinct viral strains. This double infected cell can then produce viral particles with a mixed dimeric RNA genome. Subsequently, cells infected by such heterozygous virions can obtain a hybrid provirus due to template switching during reverse transcription [15], [16]. Recombination may be important, for example, in combining resistance mutations into a single genome to achieve fully drug-resistant strains [10], [11]. However, until now the contribution of recombination to HIV-1 evolution has been predominantly shown in an indirect manner, through sequence analyses [17]–[19] and *in silico* studies [20]–[22]. It is not indisputably clear whether recombination always leads to a higher rate of adaptation. The Fisher-Muller model predicts that in asexuals (non-recombining individuals) two beneficial mutations have to be fixed sequentially, whereas recombination can combine beneficial mutations that have evolved in parallel. Furthermore, since asexual organisms have genetically linked loci, in theory they are more prone to the accumulation of deleterious mutations. On the other hand recombination may be disadvantageous since the net effect may result in breaking up favorable combinations of mutations more often than combining beneficial mutations [23]–[27]. In addition, recombination may not be an essential process, since if the mutation rate is sufficiently high and the population size large, genomes carrying multiple beneficial mutations should appear even in asexual populations [28], [29].

In this study we set out to determine whether the host-cell environment can affect the adaptability of HIV-1 *in vitro*. Two HIV-1 strains were each cultured in two different T-cell lines (MT4 and C8166) for 110 days (with six replicas for each treatment; making a total of 24 serial passage lines). Relative viral fitness was determined at regular time intervals by letting the evolved viral strains compete against a reference strain. All viruses cultured in the MT4 T-cell line increased rapidly in fitness while viruses cultured in the C8166 T-cell line did not show any increase in fitness. Through sequence analyses and infections with HIV-1 strains expressing fluorescent protein we were able to relate the lack of adaptation in the C8166 T-cell line to a decreased rate of recombination of HIV-1 in this T cell-line.

## Results

### Serial passage of two HIV-1 strains on MT4 and C8166 T-cells

To assess to what degree the host-cell environment influences the adaptability of HIV-1 we performed a large-scale selection experiment with two distinct HIV-1 strains in two T-cell lines (MT4 and C8166). The ‘wild type’ (wt) virus is the widely used molecular clone pLAI [30], whereas, the mutant (mt) virus lacks one NF-κB DNA-binding site in its Long Terminal Repeat (LTR) transcriptional promoter, and is therefore expected to have a reduced initial fitness [31]. MT4 and C8166 are two genetically distinct T-cell lines, which have been immortalized by the human T-cell-leukemia-lymphoma virus type-I [32]. We have shown previously that mutations in the transcriptional promoter have different fitness effects when tested in the MT4 and C8116 cell line [33]. This suggests that differences in cellular environment may affect the evolutionary outcome of a replicating virus. Initial relative fitness and replication efficiency were determined for both viruses in the MT4 and C8166 T-cell lines (Table 1 and Supplemental Figure S1), which indicates that viral population size and expansion were similar between cellular environments. As expected initial wt viral fitness was significantly higher than mt viral fitness in both cell lines (*two-tailed t-test*, p<0.0001). Wt and mt viruses were used to infect MT4 and C8166 T-cells to establish 24 serial passage lines (six replicates for each virus and cell line combination). Over a period of 110 days, virus was harvested from each culture at peak infection (typically after 4 to 6 days) and subsequently used to infect a ‘fresh’ batch of cells. Each time after serial passage the same initial viral density and number of host cells were used. Host cell populations typically expanded from 10^6^ to 10^7^ cells over 3–4 days, and no significant differences in growth between cell lines (non-infected and infected) were found.

**Table 1:** Initial relative fitness^a^ (±s.e.m.) of wt and mt virus

Virus	Cell type	
	MT4	C8166
wt	1.04±0.02	1.02±0.02
mt	0.83±0.02	0.81±0.03

a Fitness is relative to reference strain v13

wt>mt, p<0.0001

**Figure 1:**
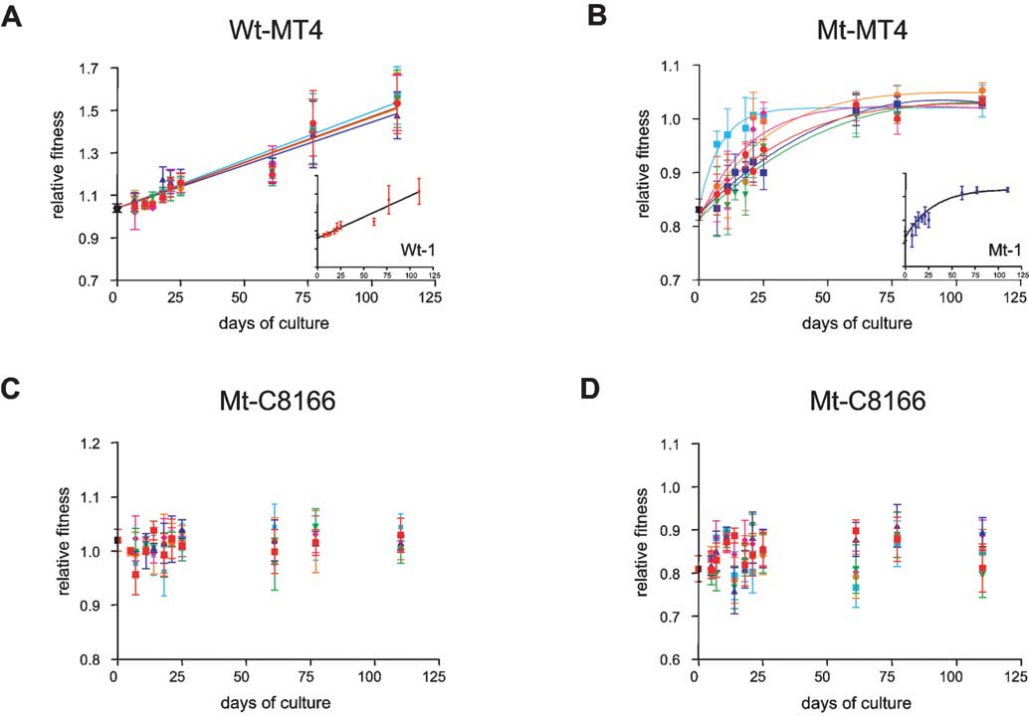
Change in relative fitness of viruses cultured on two distinct T-cell lines. A) The 6 wildtype replicates that are cultured on MT4 T-cells show a linear increase in fitness. The inset shows a single representative example of replicate wt-1. B) The 6 mutant MT4 cultured replicates show an initial fitness increase that seems to reach a maximum at around a relative fitness of 1.04±0.02. All trajectories are best described by an exponential regression model with an upper limit (F-test, p<0.05). The inset shows a single representative example of replicate mt-1 (see Table 2 for relative fitness values at day 110). C and D) relative fitness of 6 wt and 6 mt C8166 cultured viruses, respectively. Relative fitness did not significantly deviate from the starting virus over the course of the experiment (ANOVA, p>>0.05; see Table 3 for relative fitness values at day 110). Fitness is relative to reference strain v13 (also see materials and methods section.

Since the wt and mt virus have not been specifically adapted to either cell line we expected to observe a fitness increase for all serial passage lines [34]. Indeed, the six wt replicates cultured in the MT4 T-cell line (wt-MT4), increased rapidly in fitness in a seemingly linear fashion and reached a significantly higher fitness level of 1.54±0.14 over the course of the experiment (Figure 1A; Table 2, *ANOVA*, F_1,9_ = 11.276-16.350, p<0.0001). The six mt-MT4 serial passage lines initially also displayed a rapid fitness increase and they too reached a significant higher final fitness around 1.04±0.02 (Figure 1B, Table 2, *ANOVA*, F_1,9_ = 10.713-16.904, p<0.0001). However, fitness increase in the mt lines decelerated over the course of the selection experiment and seemed to reach a plateau, which was confirmed by the superior fit of an exponential model with an upper limit over that of a linear model (Figure 1B, *F-test,* p<0.05 for all replicates). The final fitness values of the mt replicates are well below that of the wt replicates (*ANOVA*, p<0.001 with Bonferroni correction). This indicates that the functional defect of the mt strain caused the virus to ‘lock’ on a lower fitness peak in the adaptive landscape [35], [36], at least for the duration of the experiment.

**Table 2:** Relative fitness^a^ (±s.e.m.) of wt and mt virus after 110 days of culture in the MT4 T-cell line

Virus and cell type of culture	Cell type-MT4			Cell type-C8166		
	Fitness	df	F^c^	Fitness	df	F
	d = 110^ b^			d = 110		
wt-1-MT4	1.53±0.14^***^	9	13.852^***^	1.31±0.05^***^	3	14.094^***^
wt-2-MT4	1.48±0.11^***^	7	12.338^***^	1.32±0.07^*^	3	7.030^*^
wt-3-MT4	1.55±0.13^***^	9	16.350^***^	1.27±0.05^*^	3	6.367^*^
wt-4-MT4	1.54±0.13^***^	9	11.276^***^	1.39±0.09^***^	3	17.873^***^
wt-5-MT4	1.54±0.14^***^	9	18.657^***^	1.22±0.03^***^	3	17.834^***^
wt-6-MT4	1.57±0.14^***^	9	14.614^***^	1.28±0.06^***^	3	7.360^*^
mt-1-MT4	1.03±0.01^***^	7	15.032^***^	1.05±0.04^***^	3	9.478^**^
mt-2-MT4	1.03±0.01^***^	9	11.405^***^	0.99±0.06^***^	3	17.638^***^
mt-3-MT4	1.03±0.01^***^	9	10.713^***^	1.03±0.05^***^	3	11.768^***^
mt-4-MT4	1.03±0.01^***^	7	16.904^***^	1.01±0.06^***^	3	12.578^***^
mt-5-MT4	1.05±0.01^***^	7	15.215^***^	1.00±0.05^***^	3	12.811^***^
mt-6–MT4	1.03±0.01^***^	7	14.925^***^	1.04±0.02^***^	3	15.496^***^

a Fitness is relative to reference strain v13

b Significance of fitness at day 110 was compared to the parental virus (wt/mt–d0) in an ANOVA containing all fitness samples measured during the experiment, p value was therefore Bonferroni-corrected for multiple comparisons

c F statistic was determined in an ANOVA with repeated measures

d.f., degrees of freedom (number of fitness samples measured during experiment); ^*^ p<0.01 ^**^ p<0.001, ^***^ p<0.0001

**Table 3:** Relative fitness^a^ (±s.e.m.) of wt and mt virus after 110 days of culture in the C8166 T-cell line

Virus after 110 days of culture	Cell type-MT4			Cell type-C8166		
	Fitness	df	F^c^	Fitness	df	F
	d = 110^b^			d = 110		
wt-1-C8166	1.02±0.01^ns^	3	2.076^ns^	1.01±0.01^ns^	9	0.530^ns^
wt-2-C8166	1.01±0.01^ns^	3	1.753^ns^	1.02±0.01^ns^	9	0.437^ns^
wt-3-C8166	1.01±0.01^ns^	3	2.842^ns^	1.00±0.03^ns^	9	0.562^ns^
wt-4-C8166	1.02±0.02^ns^	3	1.575^ns^	1.03±0.03^ns^	9	0.151^ns^
wt-5-C8166	1.00±0.02^ns^	3	1.443^ns^	1.01±0.02^ns^	9	0.062^ns^
wt-6-C8166	1.01±0.01^ns^	3	1.509^ns^	1.04±0.02^ns^	9	0.754^ns^
mt-1-C8166	0.86±0.02^ns^	1	0.620^ns^	0.82±0.06^ns^	8	1.215^ns^
mt-2-C8166	0.78±0.06^ns^	1	0.254^ns^	0.89±0.03^ns^	8	1.843^ns^
mt-3-C8166	0.79±0.06^ns^	1	0.117^ns^	0.80±0.05^ns^	8	2.164^ns^
Mt-4-C8166	0.87±0.04^ns^	1	0.995^ns^	0.89±0.04^ns^	8	1.016^ns^
mt-5-C8166	0.77±0.07^ns^	1	0.495^ns^	0.86±0.04^ns^	8	1.171^ns^
mt-6-C8166	0.78±0.06^ns^	1	0.277^ns^	0.85±0.05^ns^	8	1.188^ns^

a Fitness is relative to reference strain v13

b Significance of fitness at day 110 was compared to the parental virus (wt/mt–d0) in an ANOVA containing all fitness samples measured during the experiment, p value was therefore Bonferroni-corrected for multiple comparisons

c F statistic was determined in an ANOVA with repeated measures

d.f., degrees of freedom (number of fitness samples measured during experiment); n.s., not significant

Surprisingly, none of the twelve serial passage lines that were cultured on C8166 T-cells significantly changed their fitness (Figure 1C and, D, Table 3, *ANOVA*, F_1,9_ = 0.062-2.164, p>0.05). There are several potential explanations for this apparent lack of adaptation. First, the basic mutation rate might be low in the C8166 T-cell line as compared to the MT4 T-cell line. This, however, is unlikely since the mutation rate depends mainly on the viral reverse transcriptase enzyme, which was identical in all situations. Besides, we confirmed by sequence analysis that the basic mutation rate was similar in both cell lines (see next section). Second, there may have been fewer adaptive mutations available in the C8166 T-cell line, for instance, because the initial viral strains were already better adapted to the C8166 T-cell line than to the MT4 cell line. We were able to dismiss this possibility as it turned out that the viruses that were cultured on the MT4 line also had a significantly higher fitness on the C8166 cell-line (Table 2, *ANOVA*, F_1,3_ = 6.367-17.874, p<0.01), which illustrates that in principle adaptation to the C8166 cell line was possible. As a cross-check, we also determined fitness of the C8166 serial passage lines on the MT4 T-cell line, which demonstrates that these viruses had not changed their fitness on MT4 cells (Table 3, *ANOVA*, F_1,3_ = 0.117-2.842, p>0.05). These data illustrate that both wt and mt virus had a large adaptive potential. In spite of this potential, none of the C8166 cultured viruses had increased their fitness, neither on C8166 cells nor on MT4 cells.

### High polymorphism and linkage between genetic regions in C8166 cultured viruses

To determine whether there was a genetic basis for differences in adaptation we sequenced two domains of the HIV-1 genome; the LTR and viral Envelope (*Env*). Each of the 24 serial passage lines were analyzed by sequencing ten clones for each region and this was repeated for four time points, whereas for a fifth time point (at 61 days) only *Env* was sequenced. On average, nucleotide diversity in both cell lines was similar, which underpins our previous assumption that the basic mutation rate in both cell lines was similar (Table 4). There was no significant difference in linkage disequilibrium between MT4 and C8166 cultures, possibly due to the overall relatively low genetic variation. Sequence analysis of the LTR region showed that inactivation of the NF-κB site in the mt virus was stably present up to the end of the experiment in all serial passage lines. This confirms that the LTR ‘handicap’ remained present during the time frame of the experiment and this gives a likely explanation why the mt-MT4 replicates were ‘locked’ on a lower fitness peak compared to wt-MT4.

**Table 4:** Nucleotide diversity (π±s.e.m.)

Virus/Cell type	π
wt-MT4	0.0049±0.001^a^
wt-C8166	0.0052±0.001^a^
mt-MT4	0.0044±0.001^b^
mt-C8166	0.0037±0.006^b^

a *two-tailed t-test* p = 0.85

b *two-tailed t-test* p = 0.56

There was no obvious bias in the observed mutations; all cultures had a similar number of transitions and transversions and also synonymous and non-synonymous changes in *Env* were not significantly different between cultures (Table 5). After 110 days of culture four MT4 cultures contained a single point mutation that had gone to fixation. In three cases this mutation was located in the LTR region; two of these mutations were transitions (G 224 A and G 368 A) and the third was a transversion (G 280 C). The fourth fixed mutation, a non-synonymous substitution at the second codon position, caused a threonine-to-lysine change in *Env* at position+7133. This lack of mutations that have achieved fixation might be partly due to coexistence of beneficial mutants. Also adaptive mutations in parts of the genome that we did not sequence might have contributed to the observed increase in fitness.

**Table 5:** Transition/transversion ratio (R) in LTR and *Env* and non-synonymous (dN)/synonymous (dS) ratio in *Env* (±s.e.m.).

Virus/Cell type	R		dN/dS
	LTR	Env	Env
wt-MT4	1.489±0.147	1.3075±0.141	0.778±0.154
wt-C8166	1.729±0.144	1.776±0.147	0.831±0.248
mt-MT4	1.412±0.087	1.704±0.285	0.855±0.249
mt-C8166	1.543±0.260	1.517±0.193	0.855±0.187

**Figure 2:**
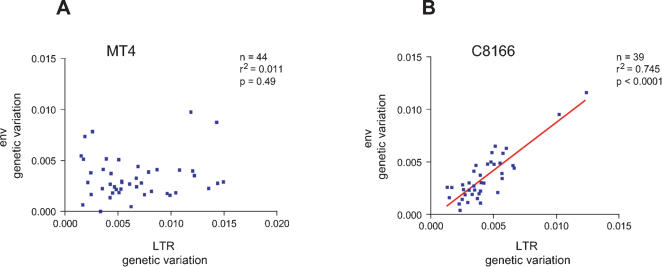
Genetic variation in the Long Terminal Repeat (LTR) and envelope region (*Env*) of MT4 and C8166 cultured viruses. For each culture, ten clones of both the LTR and *Env* region were sequenced and this was repeated for five points in time. Thus, each data point corresponds to the amount of genetic variation within a single serial passage line at a certain point in time. Genetic variation (Kimura 2 parameter) is plotted separately for the two cell-lines. A) No correlation in genetic variation in MT4 evolved viruses was observed (n = 44, r^2^ = 0.011, p = 0.49). B) There is a strong linear correlation in genetic variation between the LTR and *Env* in C8166 cultured viruses (n = 39, r^2^ = 0.745, p<0.0001). Even if the two outliers are taken out, the strong correlation remains (n = 37, r^2^ = 0.475, p<0.0001).

From the sequence analyses two distinct patterns emerge. First, plotting genetic variation for both regions for viruses cultured on the MT4 cell line reveals there is no correlation in genetic variation between the LTR and *Env* region (n = 44, r^2^ = 0.011, p = 0.49; Figure 2a), whereas there is a strong linear correlation in genetic variation between these two regions in C8166 cultured viruses (n = 39, r^2^ = 0.745, p<0.0001; Figure 2b). Even if we exclude the two outliers from Figure 2b the strong relationship remains (n = 37, r^2^ = 0.475, p<0.0001). In other words, MT4 cultured viruses show no linkage in genetic variation between the LTR and *Env* region, which is in accordance with strong selection. In contrast, C8166 cultured viruses show a strong correlation between genetic variation in the LTR and *Env* region, which is indicative of the variation being (nearly) neutral.

A second pattern emerges from the level of viral polymorphism in each culture. We observed that the polymorphism level for the wt-C8166 viruses was twice as high compared to wt-MT4 viruses (0.372±0.044 wt-C8166 vs 0.179±0.026 wt-MT4; *two tailed t-test* p<0.0001; omitting singletons from the analysis). The same pattern was present for mt viruses; the polymorphism level being twice as high in mt-C8166 viruses compared to mt-MT4 viruses (0.247±0.032 mt-C8166 vs 0.130±0.018 mt-MT4; *two tailed t-test*, p<0.001). In combination with the similar levels of nucleotide diversity between environments this implies that singletons were at least two times more common in the MT4 cultures (data not shown). These results suggest that: i) selective sweeps in the MT4 cultures have triggered a higher number of singletons in this environment [37], ii) the high polymorphism level in the C8166 cultures could be indicative for ‘clonal interference’ (see below).

### Lack of recombination in the C8166 T-cell line

We were interested in how different cellular environments may drive differences in viral adaptation. We had minimized the chance that differences in adaptation would be triggered by differences in: i) population size; both viral population size, cell population size and expansion size were similar between cultures, ii) bottlenecks; the same amount of cells were infected with the same amount of virus, and iii) mutation rate; the mutation rate of HIV-1 is (mainly) driven by the erroneous nature of the reverse transcriptase (RT) enzyme, which is produced by the virus itself. The RT gene was identical in all viruses, and consequently the basic mutation rate should be similar across viruses. We show in Table 4 that, indeed, the accumulated nucleotide diversity, which is the average number of nucleotide differences per site, was similar between the two cell types.

Nevertheless, we found a strong effect of cellular environment on adaptation, with strong fitness increase in MT4 and no fitness increase in C8166. We think this difference in adaptive potential is possibly caused by differences in recombination rate between the two cell lines. Based on the genetic analyses, the large number of singletons in MT4 cultures point to strong selection whereas the higher polymorphism level in the ‘non-adapted’ C8166 replicates might be indicative for ‘clonal interference’ between clones with marginal fitness effects competing to achieve fixation [38]–[44]. Clonal interference can put a ‘speed limit’ on the rate of evolution. Recombination can alleviate this restriction by combining beneficial mutations into a single genome and thus circumvent competition among strains [45]–[47]. A second important process that can slow down adaptation or even result in a decrease in fitness is Muller's ratchet, which can be triggered by repeated bottlenecks [48]–[51]. Although bottleneck size was the same for both environments, differences in recombination rate between environments may have caused the C8166 cultured viruses to be more affected by Muller's ratchet and/or by clonal interference between marginally beneficial mutations.

To directly test for the ‘lack of recombination’ hypothesis we assessed differences in viral recombination between MT4 and C8166 T-cells. Here we used modified HIV-1 strains encoding either the gene expressing yellow or cyan fluorescent protein (YFP/CFP) [14]. If recombination occurs between specific regions of the YFP and CFP genes these HIV-1 recombinants will express modified Green Fluorescent Protein (GFP*) which can be observed by FACS analysis [14]. MT4 and C8166 cells were infected with equal amounts of HIV-YFP and HIV-CFP (2 replicates each). Every four days (well before peak of infection) virus was harvested and a new batch of cells was infected. FACS analysis was performed after two and three rounds of serial passage (after 8 and 12 days, respectively). After eight days, over ten times more MT4 cells than C8166 cells displayed recombinant GFP* (Table 6). After twelve days the differences between cell lines had increased even further, and now almost 50 times more MT4 cells expressed GFP*. The differences in recombination correspond to differences in double infected cells (expressing both YFP and CFP). We observed 36x and 124x more double infected cells in the MT4 cultures compared to the C8166 cultures, after eight and twelve days, respectively. These data confirm that effective recombination occurs less frequently in C8166 cells than in MT4 cells, which is the result of a lower number of double infections in C8166 cells.

**Table 6:** Recombination in MT4 and C8166 cells

Cell line^a^, ^b^	8 days			12 days		
	%^c^GFP*	%YFP/CFP	CA-p24 ng/ml	%GFP*	%YFP/CFP	CA-p24 ng/ml
MT4	0.0165±0.002	0.013±0.002	101±20	0.0416±0.000	0.0423±0.014	91±9
C8166	0.0015±0.000	0.0003±0.000	82±15	0.0009±0.000	0.0003±0.0001	89±12

a On average 5.5±1.0%cells were infected on days eight and twelve.

b Every four days virus was harvested and a new batch of cells were infected.

c Percent cells either expressing GFP* or both YFP and CFP.

## Discussion

### Viral genotype can affect the evolutionary outcome

In this paper we set out to determine whether differences in the host-cell environment can drive divergent evolution of HIV-1. We tested two different virus strains, one ‘wildtype’ and a deliberately handicapped mutant, across two distinct T-cell environments. After 110 days of culture we obtained some very interesting results. First, we showed that viruses when cultured on MT4 cells evolved to a higher fitness level. Interestingly, the wild type virus showed a continual linear increase in fitness, whereas the adaptive rate of the mutant virus slowed down and a fitness plateau was reached. As a consequence, the mutant virus obtained a lower fitness level than the wildtype virus. This suggests that viruses with a small initial genetic difference can consistently evolve towards different local fitness peaks in the viral adaptive landscape [35], [36], [52]. It also underlines that the viral genotype significantly contributes to the adaptive potential. This is especially interesting in the clinical context of viruses that carry drug resistance mutations, for which the virus pays a fitness cost [53]. The virus may balance this cost by accumulating compensatory mutations [54]–[61]. Our results suggest that the adaptive potential of the resistant genotype may be diminished from that of the original wildtype. Therefore, it might be advantageous to continue treatment of an HIV-1 infected patient, even when resistance develops [61]–[65]. However, this approach needs careful consideration since cross resistance against other drugs may evolve [66] and there is at least one example of a drug resistant HIV-1 strain that evolved to a higher fitness than that of the initial wildtype strain [67].

### The host-cell environment affects the evolutionary potential of HIV-1

The most important finding in our study is that none of the twelve serial passage lines that were cultured on C8166 cells resulted in an increase of viral fitness. The subsequent sequence analyses and infections with fluorescent protein expressing viruses demonstrated a lower effective recombination rate in C8166 cells, as compared to MT4 cells. This reduced recombination rate is a likely candidate to have caused the evolutionary stagnation. Recombining populations may have several advantages over non-recombining populations in adapting to a new situation [25]–[27]. Recombination can avoid clonal interference, that is, competition between mutations of almost equal fitness increase. Furthermore, non-recombining populations may experience Muller's ratchet, which is the stochastic loss of the most fit genotype in the population due to the gradual accumulation of deleterious mutations [48]–[50]. Muller's ratchet is highly sensitive to drift and may for instance be triggered by repeated bottlenecks at serial passage. Moreover, recombination can reduce the mutational load, which is a deterministic process where deleterious mutations accumulate in the genome due to insufficient productivity of the fittest class [27], [68], [69] and background selection, where the probability of fixation of a beneficial mutation is lowered if it arises in a genetic background with low fitness [41], [70]–[72]. All these mechanisms may have contributed to our results, although it is impossible at this stage to pinpoint which effects have been most important.

It is not clear to what extent recombination contributes to the adaptability of HIV-1 *in vivo*. An *in silico* study by Althaus and Bonhoeffer [22] indicates that recombination can speed up the adaptive rate of HIV-1 if the effective population size is sufficiently large. Also, *in vitro* experiments have shown that recombination can be beneficial and can *e.g.* lead to the rapid emergence of resistance against multiple drugs [10], [11], [73]. However, the conditions in which these experiments were performed, infecting cells with equal mixtures of two partially resistant strains, strongly favour the emergence of recombinants, as the beneficial mutations are already present at high frequency. In this report we show that the host-cell environment can greatly influence the adaptive potential of HIV-1. We conclude that differences in co-infection rate and consequently the effective recombination rate provide a mechanistic explanation for these differences in adaptability. Interestingly, two recent publications demonstrate that cellular factors are involved in mediating viral recombination [14], [74]. In our experiments differences in recombination rate seem to be largely due to differences in the level of co-infection of host cells, which could be due to cell-type specific expression levels of the viral (co)-receptor or other cellular factors. These combined results raise the exciting possibility to combat retroviral infections by modulating cellular factors that reduce viral recombination and consequently the virus' adaptive potential.

## Materials and Methods

### Virus constructs

Two viruses were used in the selection experiment; the molecular clone pLAI (wt) [30] and pLAI with an inactivated NF-κB-II site in its Long Terminal Repeat (mt). The NF-κB-II DNA-binding site, which is positioned from-105 to-96 relative to the transcription start site, was inactivated by exchanging the sequence 5′-GGGACTTTCC-3′ with 5′-CCCCCCCCCC-3′ through a PCR strategy with a mutagenic primer on the pBlue3′LTR and pBlue5′LTR intermediate plasmids as described previously [75]. The mt molecular clone was obtained by insertion of the *XhoI-HindIII* fragment of pBlue3′LTR into the 3′LTR of the HIV-1 molecular clone pLAI and the *ClaI*-*XbaI* fragment of pBlue5′LTR into the 5′LTR of the HIV-1 molecular clone pLAI.

### Cell lines

The human lymphocytic MT4 and C8166 T-cell lines were cultured in RPMI 1640 (Gibco BRL) supplemented with 10%fetal calf serum, penicillin (100 units/ml) and streptomycin (100 units/ml). MT4 and C8166 are T-cell lines that have been immortalized through transformation by the human T-cell leukemia-lymphoma virus type I [32]. Although exact differences between the T-cell lines have not been assessed, several differences are known, for instance in the pool of available transcription factors such as ETS1, C/EBP, GATA, NF-kB, STAT5 and SP1 [76]–[79]. The cervix carcinoma cell line C33A (ATCC HTB31) was cultured in Dulbecco's Modified Eagle's Medium (Gibco BRL) with the same supplements. All cell lines were kept at 37°C and 5%CO_2_.

### Virus stocks and replication

C33A cells were calcium phosphate transfected with 5 µg DNA of the respective molecular clone to produce virus stocks. The virus concentration was determined by the CA-p24 Enzyme-Linked ImmunoSorbent Assay (ELISA) [75]. Replication assays were performed by infecting 10^6^ MT4 or C8166 T-cells with 1 ng CA-p24 virus stock, and virus replication was followed by measuring CA-p24 production.

### Selection experiment

Twenty four cultures were founded by infecting 10^6^ MT4 or C8166 T-cells and 1 ng CA-p24 of virus (wt or mt; 6 replicates each). Infections were monitored for viral replication by measuring CA-p24 production in the culture supernatant and by monitoring syncytia formation by microscopic inspection, with peak of infection typically reached between 4–6 days of culture. Subsequently, virus was isolated and a fresh batch of cells was infected (MOI = 0.001). The cultures were maintained up to 110 days and approximately every three days virus and cellular DNA was isolated and frozen at −80°C.

### Competition experiments

Competition experiments were performed against “virus 13” (v13) to determine relative fitness of wt and mt at the start of the culture and at specific time points during the selection experiment. v13 contains a single mutation (C to G change at position-68 relative to the transcription start site) in the Sp1 DNA binding site in the Long Terminal Repeat. The mutation served as a marker that can be tracked by sequencing, and makes the v13 virus slightly less fit compared to wt in MT4 cells, but is indistinguishable in fitness from wt in C8166 cells [33]. For each competition experiment, a total of 10^6^ cells were infected with v13 and a sample of the cultured virus of interest (0.5 ng CA-p24 each). Competition experiments were performed as previously described [12], [33] at an initial multiplicity of infection (MOI) of approximately 0.001. The relatively small number of viral generations within the competition experiments, compared to the large initial number of infected cells, ensures that *de novo* mutations during the competition experiment will not affect our fitness estimates. Even if such mutations are advantageous, they will not reach substantial copy numbers over the duration of the experiment. Direct evidence for insensitivity of our method for *de novo* mutations comes from the high reproducibility of fitness estimates between replicates and the fact that we never observed any new mutations in our sequencing [33]. Also recombination is unlikely to affect our fitness estimates because, as explained in the main text, effective recombination for HIV depends on multiple infection of cells, and as indicated the MOI in our experiments is low. Furthermore, we have never observed recombinant genotypes in our sequencing data [33]. Competitions were repeated two to three times and each competition was continued for three passages by isolating virus at peak infection and subsequently infecting a fresh batch of host cells. Total cell DNA was isolated before passage from approximately 0.25x10^6^ cells as described previously [12]. Proviral LTR sequences were PCR-amplified and sequenced with the-21M13 Big Dye Primer cycle sequencing kit (ABI) to determine the ratio of both competitors [12], [33], [80] and subsequently calculate relative fitness.

### Sequencing and genetic analyses

At specific time points (days 18, 43, 61, 77 and 110) we PCR-amplified the LTR region from position+37 to+668 and the envelope region from+7010 to+7732 (Supplemental Figure S2A and S2B). PCR fragments were subsequently cloned into a pCRII-TOPO vector (Invitrogen) and ten clones were sequenced (forward and reverse) from each region and serial passage line and for each time-point (at time point 61 only *Env* was sequenced) with the Big Dye Terminator cycle sequencing kit (ABI). In addition, we re-sequenced two time points for eight cultures to determine whether our sequencing method was accurately displaying the viral variation in each culture. Indeed, nucleotide diversity was similar between original and re-sequenced cultures, although some specific observed mutations differed. Nucleotide diversity and the transition/transversion ratio (R) were calculated with MEGAv3.1 [81] and the Kimura 2-parameter model was used to calculate genetic variation in each genetic region for each serial passage line (only time points were included when at least eight clones from each region were successfully sequenced). dN/dS ratio was calculated with DnaSP [82] The degree of polymorphism in a replicate was determined by Σ*p_i_*(1−*p_i_*), where *p_i_* is the frequency of the mutation at a specific site (singletons were excluded).

### Fitness calculation and statistics

For each competition experiment we compute the relative fitness *W_m_* of the mutant, by comparing the fold expansion of the mutant and wild type [33], [83].
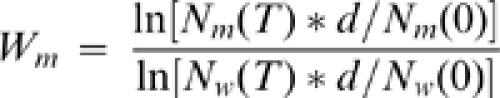
1In which, *N_m_(0)*and *N_w_(0)* are the initial densities of the mutant and wild type genotype, respectively, and *N_m_(T)*and *N_w_(T)* are the corresponding densities at the end of the experiment and *d* is the dilution factor. The relative fitness *W_m_* of a mutant is a dimensionless factor that scales the growth rate of the mutant compared to the wild type. For simplicity, we assume that mutations affect the Malthusian (*i.e.* exponential) net replication rate, as we cannot distinguish between effects on replication rate or death rate [84]. Actually, our selection regime with expanding host cell populations and serial passage transfer will tend to select for viral clones that are able to expand fast. Statistics were performed with the programs Prism 3.0 and SPSS 12.0.1.

### HIV-1 recombination assay

The genes encoding cyan or yellow fluorescent protein (CFP/YFP) are derived from pECFP-N1 and pEYFP-N1 (Clontech) and were inserted into the HIV-1 molecular clone pLAI downstream from the Env-gene and upstream of the Nef-gene. By recombination between the ECFP and EYFP genes a functional green fluorescent protein (GFP*) expressing gene is produced [14]. The detection of cells expressing fluorescent proteins was performed on a LSR-II FACS (Becton Dickinson) with a 488 nm laser equipped with standard filters or with adapted bandpass filters. 10^6^ MT4 or C8166 T-cells were infected with 10 ng CA-p24 virus stock, and virus replication was followed by measuring CA-p24 production. Due to insertion in the HIV-1 genome of the gene coding for fluorescent protein, the viruses are somewhat attenuated when compared to wt pLAI, which explains why a higher viral input was necessary in these experiments. Every four days (approximately four days before the peak of infection was reached) virus was harvested and a new batch of cells were infected. After eight and twelve days of replication 0.25×10^6^ to 0.5×10^6^ T-cells were analyzed by FACS (the experiments were performed in duplo).

## Supporting Information

Figure S1 Replication efficiency of wt and mt viruses in MT4 and C8166 T-cells. Virus production over time was followed by measuring CA-p24 in the culture supernatant. The wt virus has a clear advantage over the mt virus in both host-cell environments.
(0.78 MB EPS)

Figure S2 A. LTR sequence wt from+37 to+668. Positions are according to HXB2 numbering. B. *Env* sequence from+7010 to+7732. Positions are according to HXB2 numbering.
(0.03 MB DOC)
