# Supplementary material for: Adaptation of HIV-1 Depends on the Host-Cell Environment
Source: PLoS One. 2007 Mar 7;2(3):e271. doi: 10.1371/journal.pone.0000271 (PMC1803020; doi:10.1371/journal.pone.0000271)
Supplement: Figure S2 — A. LTR sequence wt from+37 to+668. Positions are according to HXB2 numbering. B. Env sequence from+7010 to+7732. Positions are according to HXB2 numbering. (0.03 MB DOC) [file pone.0000271.s002.doc]

40 60 80

TCCTTGATCTGTGGATCTACCACACACAAGGCTACTTCCCTGATTGGCAGAACTACACAC

100 120 140

CAGGGCCAGGGGTCAGATATCCACTGACCTTTGGATGGTGCTACAAGCTAGTACCAGTTG

160 180 200

AGCCAGATAAGGTAGAAGAGGCCAATAAAGGAGAGAACACCAGCTTGTTACACCCTGTGA

220 240 260

GCCTGCATGGAATGGATGACCCTGAGAGAGAAGTGTTAGAGTGGAGGTTTGACAGCCGCC

280 300 320

TAGCATTTCATCACGTGGCCCGAGAGCTGCATCCGGAGTACTTCAAGAACTGCTGACATC

340 360 380

GAGCTTGCTACAAGGGACTTTCCGCTGGGGACTTTCCAGGGAGGCGTGGCCTGGGCGGGA

400 420 440

CTGGGGAGTGGCGAGCCCTCAGATGCTGCATATAAGCAGCTGCTTTTTGCCTGTACTGGG

460 480 500

TCTCTCTGGTTAGACCAGATTTGAGCCTGGGAGCTCTCTGGCTAACTAGGGAACCCACTG

520 540 560

CTTAAGCCTCAATAAAGCTTGCCTTGAGTGCTTCAAGTAGTGTGTGCCCGTCTGTTGTGT

580 600 620

GACTCTGGTAACTAGAGATCCCTCAGACCCTTTTAGTCAGTGTGGAAAATCTCTAGCAGT

640 660

GGCGCCCGAACAGGGACTTGCTATAGTGTCAC

**Supplemental FigureS2A**

7020 7040 7060

TGGCAGTCTAGCAGAAGAGAGGTAGTAATTAGATCTGCCAATTTCACAGACAATGCTAAA

7080 7100 7120

ACCATAATAGTACAGCTGAACCAATCTGTAGAAATTAATTGTACAAGACCCAACAACAAT

7140 7160 7180

ACAAGAAAAAGTATCCGTATCCAGAGGGGACCAGGGAGAGCATTTGTTACAATAGGAAAA

7200 7220 7240

ATAGGAAATATGAGACAAGCACATTGTAACATTAGTAGAGCAAAATGGAATGCCACTTTA

7260 7280 7300

AAACAGATAGCTAGCAAATTAAGAGAACAATTTGGAAATAATAAAACAATAATCTTTAAG

7320 7340 7360

CAATCCTCAGGAGGGGACCCAGAAATTGTAACGCACAGTTTTAATTGTGGAGGGGAATTT

7380 7400 7420

TTCTACTGTAATTCAACACAACTGTTTAATAGTACTTGGTTTAATAGTACTTGGAGTACT

7440 7460 7480

GAAGGGTCAAATAACACTGAAGGAAGTGACACAATCACACTCCCATGCAGAATAAAACAA

7500 7520 7540

TTTATAAACATGTGGCAGGAAGTAGGAAAAGCAATGTATGCCCCTCCCATCAGCGGACAA

7560 7580 7600

ATTAGATGTTCATCAAATATTACAGGGCTGCTATTAACAAGAGATGGTGGTAATAACAAC

7620 7640 7660

AATGGGTCCGAGATCTTCAGACCTGGAGGAGGAGATATGAGGGACAATTGGAGAAGTGAA

7680 7700 7720

TTATATAAATATAAAGTAGTAAAAATTGAACCATTAGGAGTAGCACCCACCAAGGCAAAG

AGA

**Supplemental Figure S2B**
